# Elevated Bone Hardness Under Denosumab Treatment, With Persisting Lower Osteocyte Viability During Discontinuation

**DOI:** 10.3389/fendo.2020.00250

**Published:** 2020-05-15

**Authors:** Katharina Jähn-Rickert, Eva M. Wölfel, Björn Jobke, Christoph Riedel, Maya Hellmich, Mathias Werner, Michelle M. McDonald, Björn Busse

**Affiliations:** ^1^Department of Osteology and Biomechanics, University Medical Center Hamburg-Eppendorf, Hamburg, Germany; ^2^Telemedicine Clinic/Unilabs, Barcelona, Spain; ^3^MEDICOVER Berlin-Charlottenburg, Berlin, Germany; ^4^Vivantes Klinikum im Friedrichshain, Berlin, Germany; ^5^Garvan Institute of Medical Research, Bone Microenvironment Group, Darlinghurst, NSW, Australia

**Keywords:** osteoporosis treatment, denosumab, rebound fractures, treatment discontinuation, osteocytes, osteoblasts, osteoclasts, extracellular matrix

## Abstract

Denosumab is a potent osteoclast inhibitor targeted to prevent osteoporotic bone loss and thereby reduce fractures in the aging population. Recently, an elevated risk of rebound fractures following denosumab discontinuation was identified, unless patients were transitioned to an alternative antiresorptive medication. How denosumab affects the interaction of mechanosensitive osteocytes and bone quality remains unknown. We hypothesized that denosumab influences osteocyte function contributing to bone reorganization and increased fractures during discontinuation. Bone quality and osteocytes were assessed in archived iliac crest bone biopsies obtained from patients with high fracture occurrence from 2011 to 2016. Biopsies were obtained due to high fracture occurrence prior and during osteoporosis therapy from (i) patients with at least two semiannual subcutaneous injections of 60 mg denosumab, (ii) patients with rebound fractures during discontinuation, and (iii) patients of a treatment-naive group. In total, biopsies from 43 individuals were analyzed (mean age, 65.5 ± 12.1 years). Our results showed that during denosumab treatment, iliac cortical bone had a higher bone tissue hardness compared to treatment-naive bone (*p* = 0.0077) and a higher percentage of mineralized osteocyte lacunae (*p* = 0.0095). The density of empty osteocyte lacunae was higher with denosumab compared to treatment-naive (*p* = 0.014) and remained high in trabecular bone during discontinuation (*p* = 0.0071). We conclude that during denosumab treatment, increased bone hardness may contribute to improved fracture resistance. In biopsies from patients with high fracture occurrence, denosumab treatment reduced osteocyte viability, an effect that persisted during treatment discontinuation. High-resolution imaging of osteocyte viability indicates a role for osteocytes as a potential future mechanistic target to understand rebound bone loss and increased fractures with denosumab discontinuation.

## Introduction

Approximately one in two women and one in five men aged 50 years or older will experience a fracture as a result of age-related bone loss (osteoporosis), defining osteoporosis as major health burden in the growing aged population (1, 2). Age-related fragility fractures are often the first clinical symptom, yet remain difficult to manage with current treatment options, and therefore impact patient morbidity and mortality (3). An uncoupled, unbalanced bone turnover favoring bone resorption by osteoclasts over bone formation by osteoblasts forms the basis of the disease (4, 5). Pharmaceutical treatment strategies aim to counteract the low bone mineral density (BMD) and increased fracture risk by either enhancing bone-forming activity of osteoblasts or inhibiting bone-resorbing osteoclasts (6, 7).

Under physiological conditions of bone turnover, old and damaged bone matrix is removed by osteoclasts and then replaced by newly formed matrix laid down by osteoblasts. During this process, osteoblasts embed into the newly formed matrix and differentiate into osteocytes. Osteocytes reside in a fluid-filled continuum with their cell bodies inside lacunae and their dendrites inside interconnected canaliculi. The lacuno-canalicular system creates a permeability within the dense bone matrix, enabling the cellular exchange of signaling molecules. This extensive network spans throughout the bone matrix, connecting osteocytes to bone surface cells and enabling osteocytes to regulate bone remodeling activities (8–10). Osteocytes' expression of the Wnt inhibitor sclerostin limits bone formation, while osteoclast precursors are activated by the osteocytic receptor activator of nuclear factor kappa-B ligand (RANKL) (11, 12). Impaired osteocyte network connectivity through a reduction in viable osteocytes is a characteristic of osteoporosis (13). Osteocyte apoptosis has been linked to the stimulation of osteoclast-dependent bone resorption by (i) formation of apoptotic bodies containing RANKL and (ii) increased expression of RANKL by neighboring viable osteocytes (14). Furthermore, we have previously shown that in aged human bone, osteocyte lacunar hypermineralization (micropetrosis) accumulates, also impacting osteocyte canalicular signaling (15, 16). In this context, the osteocyte network in osteoporosis patients is impaired showing lower connectivity and permeability, confirming a role for osteocyte pathology in osteoporotic bone loss (17–20).

Denosumab is a monoclonal antibody against RANKL that acts as an antiresorptive agent. Thereby, the drug inhibits the differentiation of osteoclasts and limits overly active bone resorption. Furthermore, bone histomorphometry and serum analysis of bone turnover markers from denosumab- and placebo-treated patients revealed reduction in both bone resorption and bone formation (21, 22). Using denosumab as a treatment strategy for postmenopausal women with osteoporosis has successfully resulted in increased BMD as well as reduced fracture risk (23). The effectiveness of denosumab was dependent upon the skeletal site, e.g., the relative 68% reduction in spinal fracture risk is much higher than the reduced fracture risk seen at the hip (40%) (23). These promising clinical trial data have led to denosumab becoming a mainstay treatment for osteoporosis globally since 2010. Recently, however, a number of patients experienced a rebound increase in vertebral fractures following discontinuation of denosumab therapy if no other follow-up osteoporotic medications were prescribed (24–27). A possible explanation for the increased fracture risk may be found in excessive bone resorption (28). A treatment discontinuation rebound bone loss could have dramatic consequences, increasing fractures and hence morbidity and mortality; therefore, it is imperative that we determine the underlying mechanisms in order to prevent this rebound bone loss.

In this study, we aimed to comprehensively analyze *in situ* bone quality indices in iliac crest bone biopsies obtained from patients with a high fracture incidence, either under denosumab treatment or having discontinued denosumab treatment without follow-up medication, and compared to a high fracture occurrence in treatment-naive group. We hypothesized that treatment and discontinuation of treatment contribute to changes in bone quality factors and osteocyte biology. The increased bone remodeling that occurs during discontinuation reverses treatment-induced gains in bone tissue quality as well as structure. In addition, the osteocyte lacuno-canalicular network, which is of regulatory importance in bone homeostasis and bone quality, has not been sufficiently investigated during denosumab treatment nor during drug discontinuation. We postulated that with the reduced bone remodeling rate seen during denosumab treatment, tissue aging could negatively impair osteocyte viability and lead to micropetrosis (15, 16), impacting osteocyte regulation of bone turnover during denosumab discontinuation.

## Materials and Methods

### Bone Specimen and Patient Characteristics

The aim of this study was to address the question of bone quality in aged patients during and after denosumab treatment. Therefore, from 2011 to 2016, we obtained 43 iliac crest biopsies from patients consenting to biopsy analysis for research purposes. Patients underwent clinical examination according to German standardized osteology guidelines. Therefore, serum analysis, dual-energy X-ray absorption (DXA) measurements, and bone biopsies were obtained concurrently. Serum parameters were assessed in the clinical laboratory associated with the Immanuel Clinic in Berlin, Germany, adhering to local quality control standards for the clinical assessment of serum parameters. T-score measurements based on osteodensitometry at the femur and the lumbar vertebrae were performed using a DXA device (Lunar Prodigy, GE Healthcare). Bone specimens were deidentified throughout the entire study [approval by local ethics committee (Hamburg Chamber of Physicians, WF-023/19)]. The most common reason for biopsy obtainment during clinical investigations were atraumatic or nontraumatic fractures, skeletal pain, low BMD, and abnormal serum bone turnover markers. In these individuals, three groups of patients were defined: (i) nondenosumab treated with no prior pharmaceutical treatments for age-induced bone loss (treatment-naive, *n* = 11; 5 female, 6 male), (ii) denosumab treated with at least two semiannual subcutaneous injections of 60 mg denosumab (denosumab, *n* = 23; 20 female, 3 male), and (iii) denosumab treatment discontinuation without follow-up medication with a high percentage of patients experiencing rebound fractures (discontinuation, *n* = 9; 9 female). In the treatment-naive group, patients presented with either a low T-score, comorbidities affecting osteoporotic risk, fractures, or other indications for bone-specific therapy according to the DVO (the guideline for osteoporosis treatment was put forward by the umbrella organization DVO “Dachverband Osteologie e.V.”), which reflects the position of the physicians in Germany, Austria, and Switzerland and is similar to the National Osteoporosis Foundation (NOF) guidelines (29, 30). Reasons for drug discontinuation in the studied cohort included vertebral fractures, end of a clinical study, improved and stabilized BMD, or serum markers of bone turnover measuring below the desired range [physiological minima for C-terminal telopeptide of type I collagen (CTX), 704 pg/ml; bone-specific alkaline phosphatase (BAP), 5.2 μg/L]. Most patients received vitamin D supplementations. In the denosumab and discontinuation group, patients have previously been administered osteoporotic treatments such as bisphosphonates, parathyroid hormone, and estrogen or strontium ranelate. In the denosumab group, ~70% of the individuals received osteoporosis treatment prior to denosumab; whereas in the discontinuation group, ~55% of individuals received treatment before denosumab administration was initiated.

### Histological Sample Processing

Biopsies were fixed in neutral buffered 4% formaldehyde solution and further processed for undecalcified histology. Samples underwent a series of increasing ethanol solutions for dehydration, followed by infiltration and methylmethacrylate embedding as established (31). Consecutive sections of the polymerized methylmethacrylate (PMMA) blocks were cut at 4-μm thickness with a Leica microtome. Sections were stained with either Masson–Goldner trichrome, toluidine blue, or von Kossa/van Gieson.

### Histomorphological Analysis

Histological analysis was performed according to the American Society for Bone and Mineral Research (ASBMR) nomenclature guidelines (32). Sections stained with von Kossa/van Gieson were used for the histomorphometric analysis of structural bone indices. Trabecular parameters for bone structure, osteoblast, osteoclast, and osteocyte indices were performed in the central region of the cancellous bone compartment. Assessment of cortical indices for bone structure and osteocyte lacunae were performed in the entire outer cortex of the iliac crest biopsy using the Osteomeasure system and software (OsteoMetrics). Bone volume per tissue volume (BV/TV), trabecular number (Tb.N), trabecular thickness (Tb.Th), and trabecular separation (Tb.Sp) were analyzed for cancellous bone structural parameters. The outer cortex was also analyzed for mean cortical thickness (Ct.Th).

Cellular indices of bone turnover were determined on trabecular bone surfaces using the Osteomeasure system. Osteoclast parameters were first assessed by cell structure in toluidine blue stained sections, defining osteoclasts as larger multinucleated bone surface cells. Second, tartrate-resistant acid phosphatase (TRAP) activity staining was performed to determine large TRAP-positive bone surface cells. For both analyses, osteoclast number (N.Oc/B.Pm), osteoclast surface (Oc.S/BS), and eroded surface (ES/BS) were assessed to determine the bone resorption status. Bone formation indices such as number of osteoblasts (N.Ob/B.Pm), osteoblast covered bone surfaces (Ob.S/BS), as well as osteoid surface (OS/BS), osteoid thickness (O.Th), and osteoid volume (OV/BV) were assessed on toluidine blue stained sections.

### Bone Mineral Density Distribution Analysis

The bone mineral density distribution (BMDD) was measured via quantitative backscattered electron imaging (qBEI). Coplanar, polished PMMA blocks were sputter coated with carbon and visualized in an electron microscope (Leo 435 VP, Zeiss-Cambridge) in backscattered scanning electron mode (BSE Detector Type 202, K.E. Developments Ltd.) with 20 keV, a constant working distance of 20 mm, and 680 pA for beam current. The beam current was controlled by a Faraday cup (MAC Consultant Ltd. England), and gray values were calibrated on an aluminum–carbon standard. All parameters were maintained stable during imaging. The setup parameters and histogram evaluations were carried out according to our previously reported protocols (18). Quantitative evaluation of the backscattered signal intensities allowed the determination of the mineralization profiles (gray value histograms) for each specimen. The mean calcium weight percentages (CaMean), indicating the average calcium content in the cortical bone area, was measured over the whole outer cortex.

### Osteocyte Viability Assessment

Osteocyte viability is described as the number of empty lacunae (i.e., without cells) over total lacunar number (Tb eLac and Ct eLac) (33). This value was determined by staining bone sections with toluidine blue and followed by quantification of the presence or absence of osteocytes and total lacunar number (Tb.N.Lac/B.Ar). Analysis was performed using Osteomeasure software (OsteoMetrics).

We determined the role of micropetrotic osteocyte lacunae within our samples and analyzed the mineralized lacunae vs. total number of lacunae (Ct minLac) in the qBEI images as previously established (10).

In addition, a terminal deoxynucleotidyl transferase dUTP nick end labeling (TUNEL) assay on the sections with the *In Situ* Cell Death Detection Kit (Roche) according to the manufacturer's instructions was performed. Osteomeasure was used for imaging TUNEL-positive and TUNEL-negative cells in the cancellous bone compartment of the biopsies.

### Local Mechanical Properties of Bone Tissue

Local biomechanical characterization of the bone tissue was carried out using nanoindentation to confirm compositional changes of the bone matrix. Young's modulus and the local tissue hardness of the bone matrix (Poisson's ratio, 0.3) were measured (31) based on the Oliver and Pharr method (34). Therefore, PMMA blocks were polished after histological sectioning to a coplanar finish with abrasive paper (grain size P1200). Regions of interest of the outer cortical bone were indented using a Berkovich tip on the Agilent 200 nanoindenter (Keysight Technologies, Santa Rose, CA, USA). Each sample was indented 30 times with a minimal distance of 30 μm, thereby applying the continuous stiffness method.

### Statistical Data Analysis

Data were analyzed using the GraphPad Prism software 7.03. Normality of the data distribution was tested using a Kolmogorov–Smirnov test. The three study groups were compared using a one-way ANOVA with a Tukey *post hoc* test. Data were reported as mean and standard error of the mean (SEM).

## Results

### Characteristics of Patient Cohort

**Figure 1:**
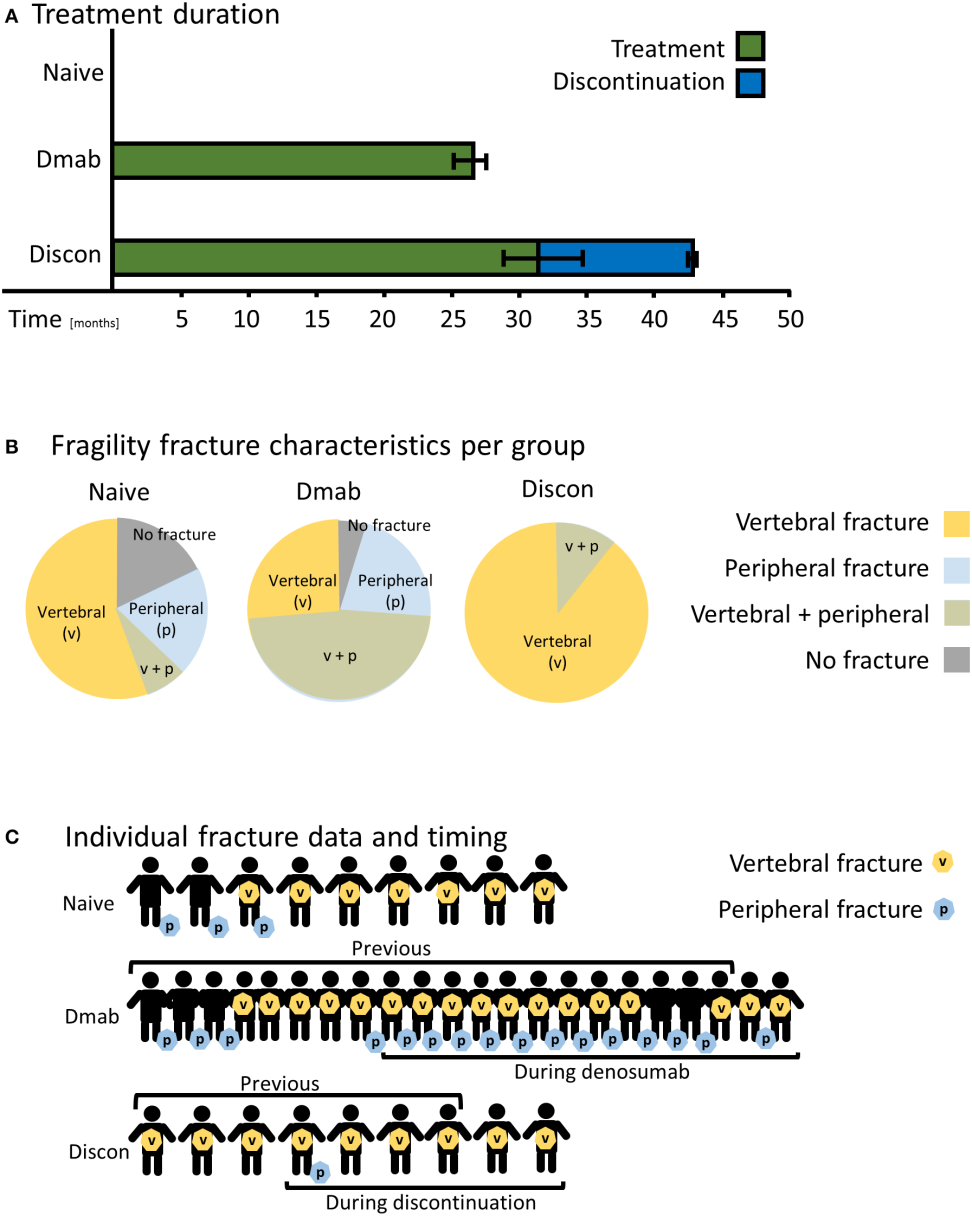
Patient characteristics based on medical records. **(A)** The average time period in months patients received denosumab (DMab, green) and the time span of discontinued denosumab treatment (Discon, blue). The time period of denosumab treatment did not significantly differ between DMab and Discon. Bar graphs show mean values ± standard error of the mean. **(B)** Fragility fracture characteristics of the study groups. Circular charts reflect the distribution of fracture types (peripheral or vertebral). **(C)** Individual fracture data including timing during denosumab treatment or during discontinuation in addition to fracture site.

No significant age differences in the study groups were evident using one-way ANOVA (Table 1). Therefore, effects of age can be ruled out in terms of bone quality characteristics in the three study groups. Figure 1A shows the time period of denosumab treatment and discontinuation.

**Table 1:** Patient characteristics evaluated based on medical records.

**Group**	**Age (years)**	**Height (m)**	**Weight (kg)**	**BMI (kg/m^**2**^)**
Naive	59.3 ± 14.8	1.7 ± 0.1	76.8 ± 12.2	25.6 ± 2.8
DMab	67.6 ± 10.6	1.6 ± 0.1	65.8 ± 18.0	25.2 ± 4.7
Discon	68.2 ± 9.0	1.7 ± 0.1	66.0 ± 9.6	23.5 ± 4.5

*Average values with standard error of the mean (SEM) for age, height, weight and BMI per group*.

While 18% of patients in the treatment-naive group and 4% in the denosumab-treatment group did not present with fragility fractures, all patients in the discontinuation group had fractured (Figure 1B). All patients in the discontinuation group suffered from spinal fragility fractures in contrast to the treatment-naive and denosumab groups in which 65% and 74% of patients, respectively, suffered vertebral fractures (Figures 1B,C). In the denosumab group afflicted with high fracture occurrence, ~60% of patients experienced a fracture during treatment. In the discontinuation group, 67% of patients experienced a rebound fracture seen as spinal fragility fractures after stopping treatment (Figure 1C).

**Figure 2:**
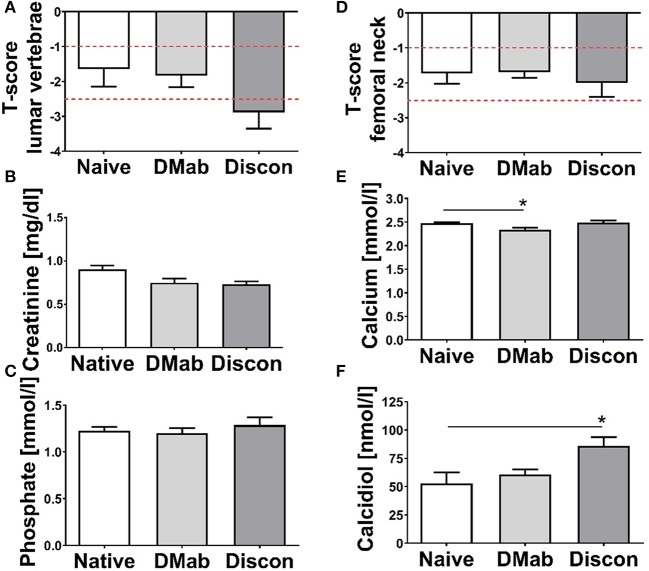
Osteodensitometry and laboratory data. T-scores were measured at the time of biopsy collection using dual-energy X-ray absorptiometry (DXA) in **(A)** the lumbar vertebrae (L1–L4) and in **(B)** the left femoral neck. Cutoff thresholds for osteopenia (−1 > T-score > −2.5) and osteoporosis (T-score < −2.5) are marked with dashed red lines. Quantification of serum parameters **(C)** creatinine, **(D)** calcium, **(E)** phosphate, and **(F)** 25-OH-Vitamin-D3 (calcidiol) taken at the time of biopsy. Levels for creatinine, calcium, and inorganic phosphate were in the physiological range. Low calcidiol levels were detected in both the treatment-naive and the denosumab group. Data presented as mean and SEM. Statistical analyses were performed using one-way ANOVA and Tukey *post hoc* test with *p < 0.05.

The T-score obtained in the lumbar vertebrae (Figure 2A) and the left femoral neck (Figure 2B) of the study groups revealed that the mean T-score of patients subjected to denosumab discontinuation was in the osteoporotic range.

While there was no difference in serum creatinine (Figure 2C), serum levels of calcium were significantly lower in the denosumab group compared to the treatment-naive group (Figure 2D; *p* = 0.044). Comparable inorganic phosphate levels were seen across groups (Figure 2E). While still in the physiological range, calcidiol level in patients on discontinuation was significantly higher compared to the treatment-naive group (Figure 2F; *p* = 0.023).

### Denosumab Discontinuation Leads to Bone Turnover Activity

**Figure 3:**
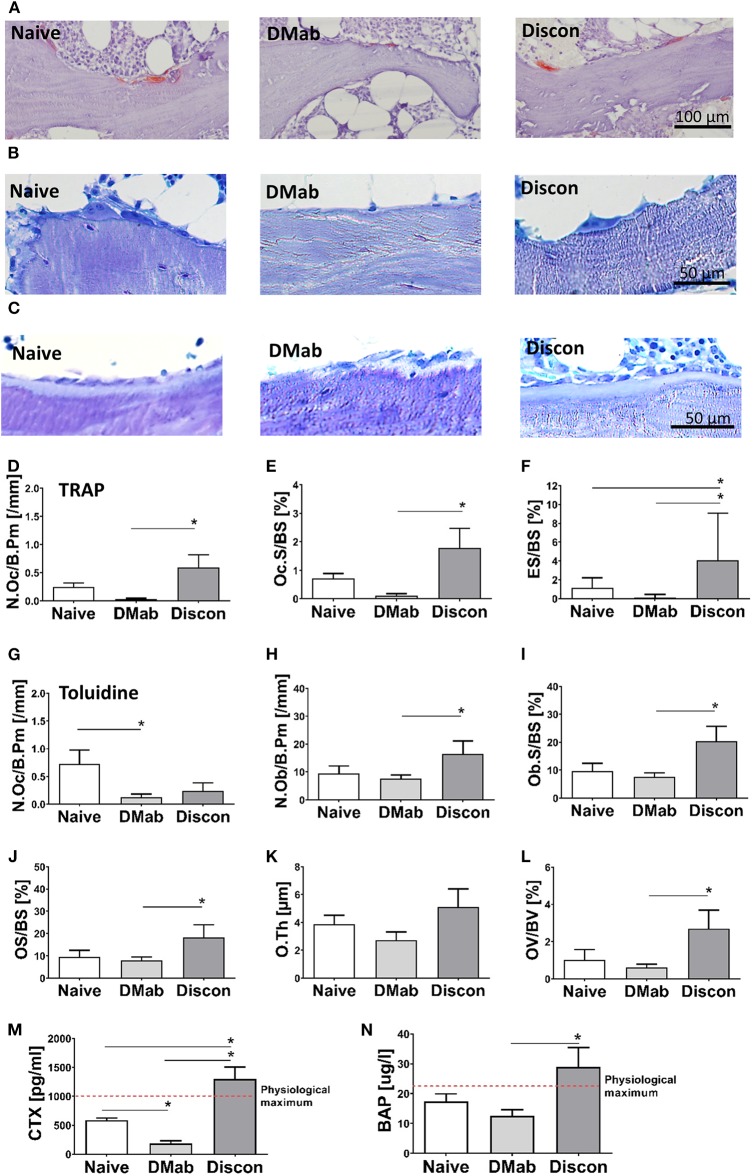
Histomorphometry and serum measurements of bone turnover markers. Osteoclast and bone resorption parameters, as well as osteoblast and bone formation parameters, were assessed in the trabecular bone compartment via static bone histomorphometry. **(A)** Representative images of tartrate-resistant acid phosphatase (TRAP)-positive osteoclasts on trabecular bone surfaces, **(B)** toluidine blue staining showing osteoclasts, and **(C)** osteoblasts. **(D–F)** Quantification of osteoclast numbers per bone perimeter (N.Oc/B.Pm), osteoclast surface per bone surface (Oc.S/BS), and eroded surface per bone surface in TRAP staining. **(G)** Quantification of osteoclast number per bone perimeter (N.Oc/B.Pm) on toluidine blue staining. **(H–L)** Quantification of osteoblasts per bone perimeter (N.Ob/B.Pm), osteoblast surface per bone surface (OB.S/BS), osteoid surface per bone surface (OS/BS), osteoid thickness (O.Th.), and osteoid volume per bone volume (OS/BV). Quantification of bone turnover serum markers **(M)** C-terminal telopeptide of type I collagen (CTX), and **(N)** bone-specific alkaline phosphatase (BAP). Graphs show mean and SEM. Statistical analyses were performed using one-way ANOVA and Tukey *post hoc* test. *p < 0.05.

Representative histological images show the presence of TRAP-positive bone surface cells, toluidine blue stained multinucleated osteoclasts and osteoid-forming osteoblasts within the collected biopsies (Figures 3A–C). TRAP-positive osteoclast number and osteoclast surface were significantly higher in the discontinuation group compared to the group on active denosumab treatment (Figures 3D,E; *p* < 0.0001; *p* < 0.0001). Eroded surface (Figure 3F) was largest in the discontinuation group compared to both the treatment-naive (*p* = 0.012) and the denosumab group (*p* < 0.0001). If osteoclasts were determined by multinucleation in toluidine blue staining, osteoclast number was significantly lower in the denosumab group compared to the treatment-naive group (Figure 3G; *p* = 0.008), and the discontinuation group did not differ statistically from both the treatment-naive and denosumab group.

The coupling of bone resorption to bone formation can lead to secondary effects when antiresorptive treatments are administered. Assessment of osteoblast numbers and osteoblasts surface did not reveal differences when comparing the denosumab group to the treatment-naive group (Figures 3H,I). However, (re)activation of bone formation was evident in the discontinuation group. Here, higher osteoblast numbers and a higher osteoblast-covered bone surface were found in comparison to the denosumab group (*p* = 0.047; *p* = 0.039). In addition, the discontinuation group had a significantly higher osteoid surface per bone surface (e.g., unmineralized bone) (Figure 3J; *p* = 0.044) along with unchanged osteoid thickness (Figure 3K) resulting in overall higher osteoid volume per bone volume in the trabecular bone region compared to denosumab treatment (Figure 3L; *p* = 0.011).

Low levels of serum marker CTX (Figure 3M) confirmed a lower bone resorption in the denosumab group compared to the treatment-naive group (*p* = 0.0018). The serum from the discontinuation patients showed CTX levels above the reference range and were significantly higher compared to the denosumab and treatment-naive groups (both *p* < 0.0001). Bone-specific alkaline phosphatase (BAP) was not only significantly altered following denosumab treatment but also elevated in the discontinuation group compared to the denosumab group and above the physiological maximum (Figure 3N; *p* = 0.0056) supporting the histological assessment of osteoblastic bone formation. Hence, bone turnover was elevated during treatment discontinuation.

### Denosumab Discontinuation Led to a Reduction in Cortical and Trabecular Bone Structure

**Figure 4:**
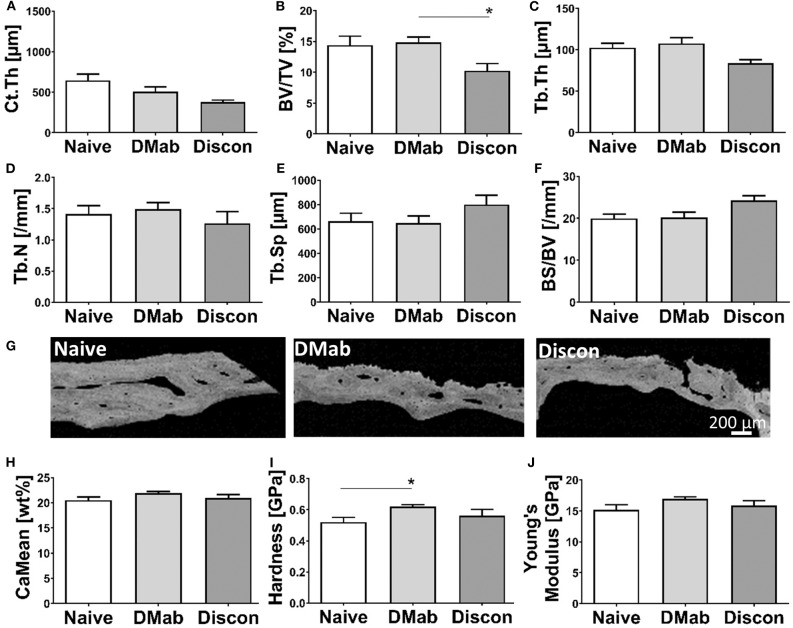
Structural histomorphometry and bone quality evaluation. **(A)** Cortical and **(B–F)** trabecular bone sections were stained by von Kossa/van Gieson and structural parameters were assessed by histomorphometry. Measurements include **(A)** Cortical thickness (Ct. Th.), **(B)** bone volume per tissue volume (BV/TV), **(C)** trabecular thickness (Tb. Th.), **(D)** trabecular number (Tb.N), **(E)** trabecular separation (Tb. Sp), and **(F)** bone surface per bone volume (BS/BV). **(G)** Representative quantitative backscattered electron imaging (qBEI) images of cortical bone. **(H)** Quantification of averaged calcium mineralization measurements from cortical bone extrapolated from qBEI images. Nanoindentation of cortical bone showing **(I)** local tissue hardness and **(J)** Young's modulus. Graphs show mean and SEM. Statistical analyses were performed using one-way ANOVA and Tukey *post hoc* test. *p < 0.05.

Interestingly, denosumab treatment did not significantly alter cortical and trabecular bone structure. With the (re)activation of bone turnover during treatment discontinuation, however, structural indices were affected. The evaluation of cortical thickness (Figure 4A) was only possible in 33 out of 43 biopsies because of incomplete cortical preservation (treatment-naive, *n* = 9; denosumab, *n* = 18; discontinuation, *n* = 6). Biopsies from patients obtained during discontinuation showed a trend for reduced cortical thickness when compared to treatment-naive biopsies (*p* = 0.052).

In the discontinuation group, a significantly lower bone volume (Figure 4B) was detected in the trabecular compartment in comparison to the denosumab group (*p* = 0.020). The changes in bone volume fraction were predominantly caused by slightly altered trabecular thickness (Figure 4C; *p* = 0.057) but not trabecular number (Figure 4D) leading to higher trabecular separation (Figure 4E). The bone surface per bone volume ratio (Figure 4F) was not significantly different between the groups.

### Denosumab Treatment Led to Higher Local Tissue Hardness

The local mechanical properties of bone can predict fracture risk (35, 36). Representative qBEI images reflecting the cortical bone mineralization show local differences in bone quality (Figure 4G). As calcium is the most abundant element in mineralized bone tissue and its amount and distribution reflects tissue hardness, we analyzed the bone mineral density distribution reflected by calcium weight percentages in the bone biopsies. The mean calcium content (Figure 4H) did not show any differences between the groups. The denosumab group showed a significantly greater resistance to indentation (hardness, Figure 4I) compared to the resistance of the treatment-naive group (*p* = 0.0077), yet this effect was reversed in the discontinuation group. The material stiffness during elastic behavior (Figure 4J) showed a trend toward higher Young's modulus in the denosumab group compared to the treatment-naive group (*p* = 0.061).

### Higher Number of Empty and Mineralized Osteocyte Lacunae Following Denosumab Treatment

**Figure 5:**
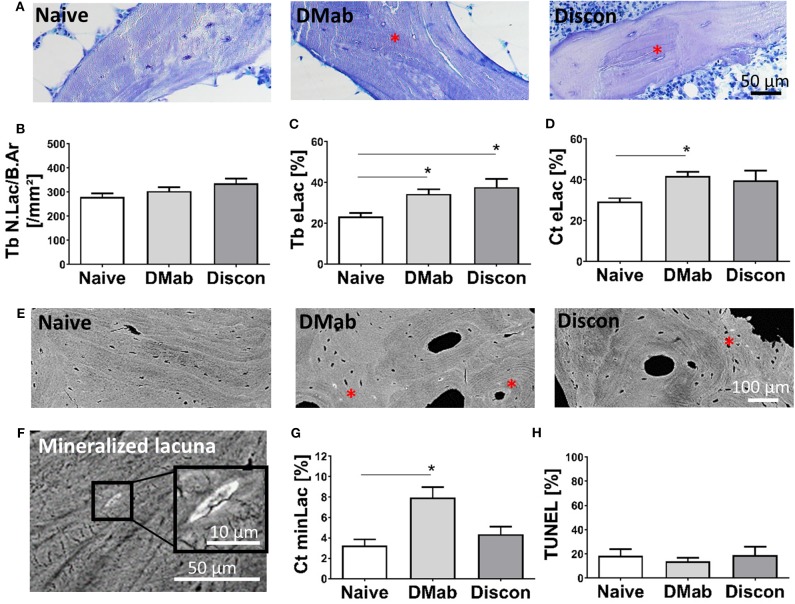
Osteocyte network characteristics. **(A)** Representative toluidine blue stained histological images of trabecular bone. Viable osteocyte lacunae with cell nuclei are evident before denosumab treatment initiation. Red asterisks designate areas of empty osteocyte lacunae not only during denosumab treatment DMab but also after treatment cessation. **(B)** Quantification of total number of lacunae in trabecular bone per bone area (Tb N.Lac/B.Ar) and **(C)** number of empty lacunae without cell nuclei in trabecular bone (Tb eLac; shown in percent). **(D)** Number of empty lacunae in cortical bone. **(E)** High-Resolution backscattered scanning electron microscopy imaging enables the identification of osteocyte lacunae subject to mineralization (i.e., micropetrosis) in cortical bone (red asterisks). **(F)** High magnification reveals lacunar occlusion with mineral. The lacunar occlusion represents the endpoint of prior osteocyte death accompanied by the growth and fusion of apoptotic bodies. **(G)** Micropetrotic lacunae were predominantly found (Ct minLac) in cortical bone when treated with denosumab, while treatment discontinuation showed a return to characteristics compatible with the treatment-naive status, where less occlusion of osteocyte lacunae was evident. **(H)** Percentage of apoptotic osteocytes in cancellous bone assessed by TUNEL assay did not vary between the groups. Graphs show mean and standard error of the mean (SEM). Statistical analyses were performed using one-way ANOVA and Tukey *post hoc* test. *p < 0.05.

Histomorphometry of osteocytes (representative images shown in Figure 5A) presented unchanged number of total lacunae per trabecular bone between the groups (Figure 5B), while the number of empty osteocyte lacunae (Figures 5C,D) without the presence of viable cells was higher in the denosumab-treated group compared to the treatment-naive group and remained high during discontinuation in the trabecular bone (denosumab vs. treatment-naive, *p* = 0.011; discontinuation vs. treatment-naive, *p* = 0.040). A similar trend was seen in the cortical bone compartment (representative qBEI images of the cortex, see Figure 5E).

One suggested endpoint of osteocyte death is the mineralization of the osteocyte lacuna (Figure 5F). Here, we determined the number of these micropetrotic lacunae in the cortical bone compartment (Figure 5G) and found the highest percentage of mineralized lacunae in the denosumab group compared to the treatment-naive group (*p* = 0.0095). The number of micropetrotic lacunae in the discontinuation group showed unchanged values (denosumab vs. discontinuation, *p* = 0.07).

To determine a potential direct interaction of denosumab on osteocyte viability, TUNEL assay was performed (Figure 5H). No significantly different level of osteocyte apoptosis was determined in the cancellous bone that could account for the increase in empty nor mineralized lacunae with denosumab and discontinuation. This result rather suggests an indirect effect of a low bone turnover rate upon denosumab treatment that prevents the adequate replacement of dying osteocytes within their bone microenvironment.

## Discussion

In this study, we present a comprehensive bone quality assessment utilizing bone biopsies obtained from (i) aged patients with high fracture occurrence under denosumab treatment, (ii) from patients whose denosumab treatment was halted afflicted with rebound fractures, and (iii) from an age-matched treatment-naive group.

All patients included in our study had a high fracture incidence despite both their treatment group and the fact that their vertebral and hip BMD T-scores were only within the osteopenic range. Pasco et al. reported in 2006 that an increasing number of women sustain fractures despite presenting with normal or osteopenic BMD or T-scores (37). Indeed, in the latter study, 50% of the fractures that occurred in the 616 postmenopausal women aged years and older were in patients with a BMD in the osteopenic range. In this context, our study provides detailed insight in the tissue characteristics of cohorts presenting with high fracture rates despite moderately changed BMD. This group of patients are challenging to diagnose and treat. It is of clinical relevance to understand the changes in bone quality at the tissue level when fractures occur during denosumab treatment but importantly also during its discontinuation. In agreement with previously reported data (21), we found a significant reduction in bone resorption in patients treated with denosumab. While in the FREEDOM trial an absence of osteoclast indices were seen in the histomorphometric data, the STAND trial, which focused on the effects of denosumab treatment following alendronate pretreatment, found a significant reduction in osteoclast indices (21). We observed a similar result in our study. Here, a reduction in multinucleated osteoclasts was found following a 24-month treatment of denosumab. We determined the resorption pattern under denosumab treatment in both the serum using CTX and at the tissue level using cellular histomorphometry. After treatment discontinuation, the bone structure was compromised with a trend toward lower cortical thickness and significantly lower trabecular bone volume. By TRAP activity in osteoclasts, we demonstrated histologically a local (re)activation of osteoclast-driven bone resorption in the iliac crest bone at the time of biopsy obtainment in the discontinuation group, and we found an elevated serum concentration of CTX above the reference range in these patients reflecting global bone resorption. By histomorphometry, we determined osteoclasts both as (i) large multinucleated surface cells in toluidine blue stained sections and as (ii) TRAP-positive bone surface cells. However, we found differing results using these two approaches with (i) lower osteoclast numbers with denosumab treatment compared to treatment-naive only visible by toluidine blue stained sections and (ii) higher osteoclast number with discontinuation compared to treatment-naive determined by TRAP activity. The CTX serum levels determined both a significantly lower bone resorption with denosumab treatment and a significant (re)activation of bone resorption. Of note, the fractures occurring in the discontinuation group were all vertebral fractures, whereas in the other groups, fractures also occurred in the peripheral regions. This is in line with our lumbar vertebral T-score and CTX data in the discontinuation group. Since our histomorphometry data were derived from the iliac crest biopsies, it might not be the ideal but only accessible location to examine the rebound effect. Other studies (24, 27) have also reported that rebound fractures during denosumab withdrawal mainly occurred in the vertebrae, which is supported by our results. The (re)activation of bone resorption after drug discontinuation has been documented in previous clinical studies (38, 39) primarily by means of bone turnover markers.

Despite the increased serum CTX and lower bone volume fraction of the trabecular bone and a tendency toward lower cortical thickness in the discontinuation group, a (re)activation of bone formation, as demonstrated through serum BAP and cellular histomorphometry, may contribute to recovery of bone mass over time. The lack of an increase in two-dimensional bone volume in the iliac crest in the denosumab group was unexpected; however, it aligns with antiresorptive agents preventing further bone loss, not rebuilding bone. Given that our treatment period was only 24 months, this finding is also consistent with previously published results of the large-scaled clinical trials (21) in which only long-term denosumab treatment of 10 years led to a significant increase in BV/TV (40).

Previous reports have demonstrated a significantly higher density of bone mineral after 2–3 years of denosumab treatment (40). While we could only determine a trend in the same direction as previously published, our high fracture risk population is small. Despite this, we demonstrated an increase in tissue hardness, meaning increased resistance to indentation, in the bone biopsies from patients with active denosumab treatment. While a lower bone tissue hardness, as seen in very high turnover conditions (e.g., in Paget's disease of bone), suggests an altered intrinsic mechanism leading to more plastic deformation (31); higher hardness in the treatment group suggests less plastic deformation, i.e., permanent deformation, possibly improving the bone's response to loading at the small-length scale. Denosumab treatment of postmenopausal women has been shown to lower fracture risks in large clinical trials (23, 41) due to increased bone mass. Our analyzed cohort with high fracture incidence did not demonstrate reduced fractures during denosumab treatment, however. This is likely since all patients had a history of multiple fractures, which was the reason for biopsy obtainment. Most patients in our cohort had sustained fractures before, during, and after denosumab treatment; bone biopsies were obtained for further histopathological assessment. In this context, the occurrence of vertebral fractures during denosumab treatment was the main reason for the decision to discontinue the denosumab treatment and consider alternative treatment options. Of note, fractures that occurred in the discontinuation group were exclusively vertebral fractures. In agreement, we found that patients in the discontinuation group had reduced lumbar vertebral T-score. This potential loss in vertebral bone mineral density and subsequent increased fracture risk has recently been described by other researchers to occur in patients who discontinued denosumab treatment without a follow-up treatment regime (42). Since 2016, several articles have reported patients suffering from rebound bone loss and vertebral fragility fractures during discontinuation from denosumab if no pharmaceutical follow-up treatment was provided (24–27). These observations caused the release of a recent statement by the European Calcified Tissue Society to object to denosumab discontinuation without close follow-ups and without a subsequent osteoporosis treatment (43). Within our high fracture cohort, our assessment of bone structural properties in patients during discontinuation supports these findings.

The osteocyte plays a key role in regulating not only bone mass but also bone quality. The present study is the first to investigate osteocyte characteristics in iliac crest biopsies obtained during and after denosumab treatment. Data on osteocytes have not been reported through clinical studies on denosumab therapy (FREEDOM and STAND). We demonstrated alterations in osteocyte network characteristics with denosumab treatment, further supporting a shift in bone quality. We showed a reduction in viable osteocytes with active denosumab treatment, which was accompanied by higher numbers of micropetrotic osteocyte lacunae. Both of which are present in bone pathologies or situations of low bone turnover indicating retention of old bone (15, 16, 20, 33). A very particular case study on aseptic necrosis of vertebral bodies (Kümmels disease) found that discontinuation of denosumab was accompanied by higher numbers of empty osteocyte lacunae (44, 45). In our study cohort, we found that 12 months postdenosumab discontinuation, the number of empty osteocyte lacunae were similar to patients with ongoing denosumab treatment and also significantly higher compared to the treatment-naive group when focusing on the trabecular bone compartment in the iliac crest. However, in the cortical bone, the number of empty lacunae was not statistically different to the treatment-naive, which might point toward a correction in percentage of viable osteocytes per bone area comparable to treatment-naive biopsies. It is difficult to speculate about the decline in viable osteocytes following denosumab treatment from our available data. The data on osteocyte apoptosis in the biopsies suggest the absence of a direct effect of denosumab on viability. Therefore, our hypothesis is that denosumab primarily induces reduction in bone turnover. Secondly, the process of physiologically dying osteocytes is not sufficiently counterbalanced by bone remodeling (active bone turnover). Hence, due to reduced RANKL activation of osteoclasts to remove the old and damaged bone matrix, dead osteocytes accumulate as they are not replaced by newly embedded osteoblasts/preosteocytes.

*Study Limitations*. While the scientific investigation of real-life patient biopsy material is informative, certain limitations are present in our study. Our cohort of patient samples is limited in size compared to large clinical trial cohorts. Furthermore, as this was not a clinical trial and instead an assessment of obtained clinical samples, all biopsies despite the treatment group were from patients with high fracture incidence; therefore, it does not reflect the typical denosumab-treated patient population. In addition, a potential sex-dependent effect cannot be anticipated in the discontinuation group. Most of the patients in the denosumab treatment groups did receive other osteoporosis medications previously, including bisphosphonates, estrogen, strontium ranelate, or parathyroid hormone. An impact of these other treatments on our study outcomes cannot be ruled out entirely. We also like to point out that while denosumab discontinuation reportedly led to high vertebral fracture incidences, our study material includes iliac crest bone biopsies and blood serum from those patients. In this study, we carried out experimental bone quality analyses exclusively in iliac crest biopsies. Cellular, structural, and compositional data are likely to be dependent on the skeletal site, and data obtained from the iliac crest may not specifically reflect bone quality in the spinal column (18). Due to the analysis of single biopsies, a longitudinal evaluation of local tissue bone turnover was not possible. Despite these limitations, our study has revealed new important insight into the processes of rebound bone loss and increased fracture rates in patients ceasing denosumab therapy.

We have confirmed the effects of denosumab treatment reducing bone resorption while further demonstrating higher tissue hardness. These effects were reversed rapidly in patients on denosumab discontinuation, with bone turnover elevated and bone structure and quality compromised. Importantly, our assessment of osteocyte viability and morphology identified an impaired osteocyte network following denosumab treatment, which was reversed in cortical bone during drug discontinuation, likely due to elevated bone turnover and replacement of empty osteocyte lacunae. A functional osteocyte network is central to the bone's mechano-dependent bone remodeling, whereas elevated levels of osteocyte cell death with subsequent occlusion of the osteocyte lacunae have been shown to endanger bone quality (16, 18, 20, 33). Hence, our investigation of osteocytes in this cohort provides new knowledge to determine the mechanisms behind this treatment-induced pathology. The contribution of this denosumab-associated osteocyte pathology to rebound bone loss upon cessation however requires further investigation: (i) to identify the direct or indirect mechanisms of denosumab treatment on osteocyte cell death and to determine the treatment duration after which it occurs, (ii) to determine whether rebound fractures are the functional outcome of this disrupted osteocyte network in denosumab-treated bone, and (iii) to identify osteocyte apoptosis prevention strategies as an approach to prevent rebound bone loss. All of these should aim to optimize treatment regimens of denosumab in patients with osteoporosis and other bone diseases.

## Data Availability Statement

All datasets generated for this study are included in the article/supplementary material.

## Ethics Statement

The studies involving human participants were reviewed and approved by Hamburg Chamber of Physicians. The patients/participants provided their written informed consent to participate in this study.

## Author Contributions

BB and BJ: study design. KJ-R: histomorphometry. EW: biomechanical testing. KJ-R and EW: anonymized clinical data summary, statistical analysis. CR: qBEI imaging. MH: patient treatment, clinical data collection, biopsy obtainment. MW: histopathological biopsy analysis. KJ-R, EW, and BB: manuscript drafting. KJ-R, EW, BJ, MM, and BB: manuscript revision. All authors: data interpretation.

## Conflict of Interest

The authors declare that the research was conducted in the absence of any commercial or financial relationships that could be construed as a potential conflict of interest.
